# Knowledge and Behaviours towards Immunisation Programmes: Vaccine Hesitancy during the COVID-19 Pandemic Era

**DOI:** 10.3390/ijerph19074359

**Published:** 2022-04-05

**Authors:** Marco Dettori, Antonella Arghittu, Paolo Castiglia

**Affiliations:** 1 Department of Medical, Surgical and Experimental Sciences, University of Sassari, 07100 Sassari, Italy; madettori@uniss.it (M.D.); castigli@uniss.it (P.C.); 2 University Hospital of Sassari, 07100 Sassari, Italy

**Keywords:** vaccine hesitancy, emotional epidemiology, outrage management, SARS-CoV-2, COVID-19

## Abstract

Assessing knowledge, attitudes and behaviours towards vaccination is a key strategy when implementing national and international immunisation programmes aimed at improving compliance among the population and thereby increasing vaccination coverage. While vaccination’s role as a powerful life-saving weapon in the fight against infectious diseases has been further highlighted following the introduction of the Coronavirus Disease 2019 (COVID-19) vaccine, there is still a discrepancy between the scientific evidence on the effectiveness of vaccines and the perception of the risk attributed to them. Known as “Vaccine Hesitancy” (VH), this phenomenon is the delay in acceptance or refusal of vaccines, despite the availability of services. VH can be found in at least 15% of the worldwide population, and even professional groups tasked with promoting vaccination as a primary prevention measure, e.g., healthcare workers (HCWs), sometimes have doubts regarding vaccination. Since 2014, this Public Health problem has been increasing in 90% of countries worldwide, to the extent that in 2019 it was listed as one of the ten greatest threats to global health by the World Health Organization (WHO). VH has also affected COVID-19 vaccination, hampering the achievement of desired vaccination coverage. Monitoring this trend by studying people’s behaviour and attitudes could be a useful tool to aid Public Health, in orienting vaccination policies and designing new health education and continuous training interventions, aimed at both the general public and accountable cohorts, such as HCWs.

## 1. Background

The history of vaccines is perhaps one of the richest and most engaging chapters in the history of medicine [1,2]. From the implementation of the first smallpox vaccine in 1796 by Edward Jenner, up to the present administration of the anti-COVID-19 vaccine, millions of lives have been saved worldwide. The success of vaccinations has enabled the eradication of infectious diseases, such as smallpox, drastically reduced the incidence of debilitating diseases, such as polio and measles, and even prevented the onset of certain cancers, including liver and cervical cancer, thereby helping to increase the life expectancy of the world’s population [3,4].

According to the WHO, vaccinations prevent up to 3 million deaths per year worldwide, yet there is still a discrepancy between the scientific evidence of their validity and the perception of the risk attributed to them. This negatively affects adherence to immunisation programmes [5]. It is well known that risk perception may increase or decrease an individual’s willingness to accept vaccination, especially among certain groups (e.g., frail individuals and HCWs), since the self-perceived risk of contracting a disease is directly linked to the choice to undergo vaccination [6,7]. Moreover, since people no longer have experience of those infectious diseases that have been averted through vaccination, parents do not perceive the need to immunise themselves and their children, thus, exposing themselves to the possibility of contracting the natural disease and/or its sequelae [8,9]. The already precarious decision-making balance inherent in vaccine acceptance is also strongly influenced by what one reads online. This is particularly clear in the context of the current pandemic, in which the often inexperienced user is continuously exposed to the media’s huge volume of apparently conflicting news, including discrepancies in the disease’s impact, in terms of morbidity and mortality in different countries and in different time periods, and the opposing opinions on the effectiveness of the various vaccines available. This has made it difficult for people to find reliable and trustworthy sources of information, further increasing their sense of disorientation and confusion [10,11].

In this regard, several studies report that anti-vaccination groups have improved their communication techniques by exploiting new Information and Communication Technologies (ICTs), i.e., blogs, websites and social media, to spread their theories, thus, helping to propagate VH. This phenomenon, fuelled not only by the longstanding scepticism towards vaccination but also by the incorrect information prevailing on the Web, now affects at least 15% of the general population, exerting a negative influence on perceptions, attitudes and assumed behaviours [12]. The definition of VH developed by the WHO’s Strategic Advisory Group of Experts (SAGE) on Immunization also includes the heterogeneous group of individuals defined as “hesitant”, who lie somewhere between those who fully adhere to vaccinations and those who refuse them due to determinants, such as contextual influences, individual and group influences or perceptions related to individual vaccines and the organisations responsible for providing them [12]. In the context of social and health care, HCWs are not exempt from the phenomenon of VH, which is also present in hospital settings. This is all the more worrying, in view of the fact that VH in this cohort of individuals can have a twofold negative effect: on the one hand, it exposes HCWs to the possibility of contracting the natural disease and transmitting it to patients, and on the other, it does not allow HCWs to act consciously to counteract the false beliefs that lead to erroneous behaviour, in opposition with the principles on which the healthcare profession’s mission is based. For example, HCWs’ scepticism towards vaccination is particularly evident for certain vaccines, such as the anti-flu vaccine, whose benefit in terms of reducing the incidence, mortality and costs associated with the disease is widely underestimated, even among HCWs [13,14]. Several studies in the literature describe how, despite the fact that vaccination is offered actively and free of charge, the influenza vaccination coverage among HCWs continues to remain far below the minimum targets set [6,12,13,14,15]. A recent survey conducted by authors at an Italian university hospital also confirms some of the determinants of VH described in the literature for this population cohort: confidence, complacency and, in particular, convenience [6]. In fact, the authors’ survey showed that the most hesitant are precisely those who spend the most time in contact with patients, especially in the surgical area, and who would prefer to be vaccinated directly on the ward and for this to be made compulsory.

In order to counter this phenomenon, it will be crucial to promote communication/information strategies tailored to healthcare workers, in addition to adequate vaccination supply models (e.g., ward-based vaccination) [6,13,14,15,16].

In this context, the primary goal of Public Health is to promote health, generating scientific and professional maturity in HCWs and providing the necessary tools to build critical knowledge in full autonomy of judgment, both among colleagues and in the general population [15].

The COVID-19 pandemic has also highlighted how, among the many emotional levers that influence people’s attitudes and behaviour towards immunisation programmes, the lack of knowledge or availability of a remedy plays a decisive role in the field of vaccination. This is the basis of the phenomenon known as “Emotional Epidemiology”, described for influenza A pandemic (A(H1N1)pdm09), whereby, when a problem, such as the spread of a pandemic virus emerges, outrage increases proportionally. This leads the population to request, and sometimes demand, an effective remedy to safeguard their health (i.e., a vaccine). However, as soon as the remedy becomes available, the risk perception decreases, with a resulting negative impact on health choices that is immediately reflected in health outcomes (e.g., a decline in vaccination coverage) in all population cohorts [16]. This phenomenon has also been observed for anti-COVID-19 vaccines and, despite their demonstrated efficacy and effectiveness, more than a year after the start of the mass vaccination campaign, and the basic cycle and booster dose having been accepted by a large percentage of the population, even today, a significant proportion in all countries categorically refuses the vaccination.

The date 27 December 2020 marked the official start of the anti-COVID-19 vaccination campaign in Europe. Italy’s mass vaccination campaign was structured around a vaccination offer in line with local epidemiology, favouring methods and priorities that took into account the disease risk, the types of vaccines approved and their actual availability [17]. On the basis of the scientific studies available, increased age and the presence of comorbidities were the main variables correlating with COVID-19 mortality [17]. Therefore, the order of priority for the administration of the vaccine was as follows, with regard to age: (i) individuals aged >80 years and highly fragile individuals from February 2021; (ii) those aged 60–79 years from April 2021; (iii) those aged 19–60 years from May 2021; (iv) adolescents aged 12–19 years from August 2021; (v) children aged 5–11 years from January 2022. In addition, regardless of age, in several periods and varying from region to region, depending on the difficulties in supplying vaccines, in terms of the structure and management of vaccine hubs in the territory and the availability of resources, the following categories were also deemed priorities: (i) health personnel working in hospital and social care systems; (ii) school and university personnel, both teaching and clerical/maintenance staff; (iii) the armed forces, police and emergency services; (iv) prison services.

The breakdown of vaccination coverage recorded in Italy from the starting date of the mass vaccination campaign by age group to 11 February 2021 is shown in Table 1. 

In total, more than 135 million doses have been administered. Of these, 90% were mRNA vaccines and 10% adenoviral vector vaccines.

Table 1 shows that, although Italy has demonstrated great compliance to this vaccination, coverage for the basic cycle (i.e., two doses) in the over-12 age group falls short of the desired rate, ranging from 94.5% in the over-80s to 80.5% in the 12–19 age group, with over 3.6 million individuals who have not received any dose at all [18]. This may be justified, in part, by the fact that the over-80s cohort was identified as a priority in planning the Italian vaccination campaign, while the 12–19 cohort was dismissed as lowest in priority, and vaccination for the childhood cohort aged 5–11 years only very recently introduced. Nonetheless, recent studies have shown that emotional factors and risk perception have also influenced vaccination compliance towards COVID-19 vaccination in Italy. Indeed, determinants, such as the increasing familiarity with this disease over the last 2 years, and the full availability of vaccines and booster doses—typical factors of Emotional Epidemiology—have negatively affected vaccination compliance and continue to do so [15].

Furthermore, Emotional Epidemiology even works for rare diseases, such as meningitis. In fact, diseases with high case-fatality rates and epidemic potential recall ghosts of the past and trigger collective defence mechanisms, whose dynamics are consistent with the transmissibility of the same infectious agents [19,20]. In these cases too, the high level of outrage leads to a demand for mass vaccination by the public, as soon as there is even a single episode, only to see the same public quickly lose their perception of the risk soon afterwards. This can be seen, for example, in the meningococcal vaccination coverage in adolescence recorded in Italy, which, despite its availability, continues to be very low, thereby confirming the poor compliance among the reference target [21]. This shows that, as with immunological memory with regard to SARS-CoV-2, the memory of scientific data also tends to decline over time due to emotional factors. This illustrates why institutions must also administer booster doses of medical–scientific messages, conveyed correctly and appropriately [19].

As such, if they are to improve knowledge and attitudes towards vaccination, Public Health must understand the social, demographic and psychological determinants of VH, in order to reach the population groups most likely to refuse the vaccine, and consequently, implement strategies and activities that favour compliance towards conscious adherence. Furthermore, the language chosen to relay a health message plays a determining role in how the vaccine is accepted, as do the communication strategies or media chosen to convey it. To do this, the alignment of all the authorities involved in health communication, in producing clear and coherent messages, should be consolidated. This implies that current and future vaccination campaigns should focus on the centrality of the individual and include actions aimed at improving Health Literacy and increasing individuals’ engagement with the health system. Actions must be mainly structured around proper communication of the medical, scientific and social value of vaccination, while also building relationships based on trust, awareness and responsible action [22].

Moreover, as both the COVID-19 pandemic and the continuation of the mass vaccination campaign are evolving in real time, communication methods must be readily adaptable to ensure that the most up-to-date evidence is spread through the sources of information. To this end, bearing in mind the great potential offered by the Internet in health information research processes, health institutions have also chosen to make use of the digital communication channels. Such channels are fundamental to ensure the dissemination of medical–scientific knowledge among user-patients, with the aim of: (i) increasing the spread of high-quality health information; (ii) involving citizens/patients, making them as responsible as possible for their own health; (iii) increasing awareness about infectious diseases and the health treatments available to prevent them [21,22,23]. A good example of this is the Italian ‘Vaccinarsì’ network [24], which is a harbinger of evidence-based messages relayed in consistent terms.

## 2. Conclusions

While vaccination has been universally recognised as one of the best strategies to increase longevity and improve quality of life over the last centuries, coverage rates often fall short of the levels recommended to reduce the spread of, and to eradicate, vaccine-preventable diseases. In the next decades, one of the main challenges facing Public Health, and vaccinology in particular, is VH among the general public [25]. In the current pandemic, in which close attention must be paid to the complexity of communication processes necessary for vaccination compliance, listening forms the basis for clear and effective communication with users. Actively listening to users and their concerns constitutes a real opportunity for reflection, analysis and planning for all the actors involved in health care. In this sense, while this health emergency has thrown health organisations into crisis by highlighting gaps and delays in care, it has also confirmed the need to bolster measures aimed at improving the health system’s ability to respond to a possible future crisis situations. In this context, a speedy digital and organisational transformation is pivotal. Moves should be made towards a connected care model, built on the patient’s needs and oriented to the continuity of care and to the community it serves [26,27,28].

In fact, research shows that preventive healthcare interventions must be flanked by well-structured information and communication campaigns, implemented through all available channels, including ICTs, if they are to produce real improvements or to prevent negative changes in terms of health behaviour [23].

It follows that, in order to counter the phenomenon of VH, it is indispensable to promote communication and information strategies aimed at the population, with particular reference to fragile cohorts or HCWs. Such strategies could include: forging multidisciplinary alliances between healthcare providers, medical and scientific communications on vaccination; sharing and providing data on the evidence of vaccine efficacy and safety; implementing innovative communication strategies; increasing opportunities for dialogue and counselling on vaccination. International and national health authorities and agencies must work together in adopting these measures, in order to improve knowledge, attitudes and behaviours towards immunisation programmes, considering the COVID-19 pandemic era.

## Figures and Tables

**Table 1:** Number and percentages of COVID-19 vaccination by age groups in Italy up to 11 February 2022. Source: Authors’ elaboration from Presidenza del Consiglio dei Ministri (Office of the Prime Minister) report [18].

Age Groups	Population (a)	1st Dose (b)	% 1st Dose (b/a)	Single Dose (c)	Recovered (d)	% (b + c + d)/a	2nd Dose (e)	Vaccinated (c + e)	% (c + e)/a	Booster (f)	% * (f)	Awaiting 1st Dose (g)	% (g/a)
**>80**	4,594,071	4,329,833	94.3	112,339	18,802	97.1	4,230,789	4,343,128	94.5	3,894,111	92.0	243,409	2.9
**70–79**	6,016,425	5,401,790	89.8	313,664	41,315	95.7	5,295,672	5,609,336	93.2	5,101,201	94.3	259,656	4.3
**60–69**	7,532,302	6,361,805	84.5	681,949	104,899	94.9	6,208,509	6,890,458	91.5	6,055,845	93.4	383,649	5.1
**50–59**	9,645,296	8,035,467	83.3	736,513	244,758	93.5	7,796,666	8,533,179	88.5	6,957,435	89.8	628,558	6.5
**40–49**	8,781,291	7,125,115	81.1	512,269	270,431	90.0	6,923,603	7,435,872	84.7	5,322,066	81.7	873,476	10.0
**30–39**	6,790,908	5,631,064	82.9	380,114	215,770	91.7	5,432,991	5,813,105	85.6	3,644,952	75.6	563,960	8.3
**20–29**	6,029,273	5,234,190	86.8	362,727	159,336	95.5	5,048,743	5,411,470	89.78	3,306,083	72.5	273,020	4.5
**12–19**	4,620,379	3,759,039	81.4	191,533	241,634	90.7	3,525,927	3,717,460	80.5	1,517,430	55.2	428,173	9.3

* % of the population potentially targeted for an additional or booster dose who have completed their vaccination cycle for at least four months.

## Data Availability

The data presented in this study are available on reasonable request from the corresponding author.
